# Macroscopic Observation of Soil Nitrification Kinetics Impacted by Copper Nanoparticles: Implications for Micronutrient Nanofertilizer

**DOI:** 10.3390/nano8110927

**Published:** 2018-11-08

**Authors:** Allison Rick VandeVoort, Yuji Arai

**Affiliations:** 1 Department of Biological and Environmental Sciences, Georgia College & State University, Campus Box 081, Milledgeville, GA 31061, USA; allison.vandevoort@gcsu.edu; 2 Department of Natural Resources and Environmental Sciences, University of Illinois at Urbana-Champaign, Urbana, IL 61801, USA

**Keywords:** copper nanoparticles, nanofertilizer, soil, nitrification, nitrification kinetics, toxicity

## Abstract

The potential agricultural use of metal nanoparticles (NPs) for slow-release micronutrient fertilizers is beginning to be investigated by both industry and regulatory agencies. However, the impact of such NPs on soil biogeochemical cycles is not clearly understood. In this study, the impact of commercially-available copper NPs on soil nitrification kinetics was investigated via batch experiments. The X-ray absorption near edge structure spectroscopy analysis showed that the NPs readily oxidized to Cu(II) and were strongly retained in soils with minimum dissolution (<1% of total mass). The Cu^2+^ (aq) at 1 mg/L showed a beneficial effect on the nitrification similar to the control: an approximately 9% increase in the average rate of nitrification kinetics (*V_max_*). However *V_max_* was negatively impacted by ionic Cu at 10 to 100 mg/L and CuNP at 1 to 100 mg/L. The copper toxicity of soil nitrifiers seems to be critical in the soil nitrification processes. In the CuNP treatment, the suppressed nitrification kinetics was observed at 1 to 100 mg/kg and the effect was concentration dependent at ≥10 mg/L. The reaction products as the results of surface oxidation such as the release of ionic Cu seem to play an important role in suppressing the nitrification process. Considering the potential use of copper NPs as a slow-release micronutrient fertilizer, further studies are needed in heterogeneous soil systems.

## 1. Introduction

With the rise of nanotechnology within the past decade, nanofertilizers have been considered for use in agricultural fields [1]. While this technology continues to advance, the possibility for slow-release micronutrients resulting from the nanosized solid state of these products is appealing for some agricultural systems. In particular, hydrological regimes impacted by climate change could alter the mobility of micronutrients, influencing the plant growth and microbially-mediated biochemical cycles of nutrients (e.g., N and P). The advent of nanotechnology could increase the feasibility of the long desired agricultural goal of slow-release fertilizers, which are both more cost-efficient and environmentally sound [2,3]. The physical state of nanoparticles (NPs) as nanosized solid metal rather than dissolved ions has the potential to allow for a controlled release over time in soil solutions. Trace metals such as Cu and Zn, essential micronutrients for crops and microbial growth [4], are commonly produced NPs. Like many micronutrients, these metal-based NPs have the potential to be beneficial to plants and/or microorganisms by preventing deficiency; however, an overdose of metal NPs can lead to toxicity. Benefits of metallic Cu(0)NPs (CuNPs) to plants include increased shoot: root ratio in lettuce seedlings [5]. However, CuNPs can cause unique adverse reactions in plants not accounted for by ionic copper. For instance, seedlings of mung beans and wheat grown on a CuNP-impregnated agar exhibited diminished root and shoot length beginning at concentrations of 200 mg/L [6]. Metallic CuNPs are known to cause less oxidative stress on plants than free copper ions; plants take advantage of this fact by synthesizing metallic CuNPs through Cu^2+^ reduction in the rhizosphere [7]. In a study of hydroponic zucchini plant growth in the presence of CuNPs at 1000 mg/L, Stampoulis and coworkers found that exposure to metallic CuNPs resulted in a slower rate of plant growth compared to the control. Interestingly, they found that 1000 mg/L of bulk Cu had approximately the same effect as 10 mg/L CuNO_3_, and that 1000 mg/L of CuNPs had a similar effect as 100 mg/L CuNO_3_ [8]. This provides further evidence that NPs do not have the same oxidative impact on plant growth as ionic micronutrients. Other metallic micronutrient NPs have shown similar results. At 2000 mg/L, zinc NPs almost completely inhibited root growth of radish, rape, ryegrass, lettuce, and cucumber seedlings with a lesser, but still significant, inhibition of corn seedling root growth [9]. This same study also investigated aluminum NPs, which only diminished root growth in corn, and showed no effect on the other plant seedlings [9]. More recently, Gao and coworkers [10] investigated the effects of CuO NP amendments (500 mg/kg) to wheat plants. They found that aging of CuO NP enhanced the NP toxicity due to enhanced dissolution during the growth period and affected the rhizosphere biochemical conditions such as pH and the production of root exudate. Du et al. [11] studied the effect of metallic CuNP (0–200 mg/kg) on the agronomical and physiological parameters of soil grown oregano. While all CuNP treatments decreased the content of starch and sugar in leaves, the biomass of roots and shoot was increased. They reported CuNPs did not exhibit significant toxicity in oregano.

While the direct impact of metal-based NPs on soil bacteria has not been extensively studied, CuNPs have been shown to affect the growth of common environmental bacteria including *Escherichia coli* and *Staphylococcus aureus* in a pure culture environment, especially at concentrations in excess of 2% *w*/*w* (20,000 mg/kg) [12]. Although silver NPs are well-known for their toxicity to microorganisms [13,14,15], CuNPs were shown to impact the survival of the common environmental bacteria *E. coli* and *Bacillus subtilis* at slightly lower concentrations (60 mg/L) than AgNPs (70 mg/L) [16]. It has been documented that CuNPs are likely available to microorganisms in soil environments. A study by Kumar and coworkers documented a significant difference in substrate utilization compared to the control system by an arctic soil bacterial community when CuNPs were present at 66 mg/kg, indicating a shift in the bacterial community even at this relatively low concentration. This change was not at marked as the change in substrate utilization caused by the same concentration of AgNPs [17]. The impacts of other nano-micronutrients have been poorly investigated. Aluminum NPs appeared to have minimal, if any, impact on the metabolic activity of *Vibrio fischeri* [18]. In a study of metal oxide NPs, both titanium dioxide and Zn oxide NPs negatively impacted the soil bacterial community of a grassland, as demonstrated through decreased genotype richness, substrate induced respiration, and extractable soil DNA over time periods up to 60 days [19]. These metal oxide NPs may induce more oxidative impacts than metallic, zero-valent metal NPs.

While these studies suggest both beneficial and toxicological effects of metal NPs, it remains difficult to extrapolate such results to evaluate the potential use of metal NPs as nanofertilizers since agricultural soils are often ignored in the most of laboratory studies. This study aims to investigate the effects of metallic CuNPs as a nanofertilizer component on the complex nitrogen cycle in agricultural soils. The nitrogen cycle in soil systems is essential to the growth of successful crop species. In particular, the nitrification process is of particular importance. In the objective of this study was to examine the effect of CuNPs to the soil nitrification kinetics using batch biogeochemical experiments. Metallic Cu(0)NPs were chosen as a model CuNP. One can expect that dissolution and sorption of CuNPs in soils that control the bioavailability of Cu to soil bacteria. For this reason, the dissolution experiments of CuNPs were performed in conjunction with adsorption isotherm experiments of Cu^2+^ (aq) and CuNPs onto soils.

## 2. Materials and Methods

### 2.1. Materials

Fresh surface sandy loam soil from the Toccoa series (coarse-loamy, thermic typic Udifluvents) was used in this study, sourced from the Organic Farm in Clemson, South Carolina, USA. Soil was maintained at field capacity for nitrification experiments. For sorption experiments, soil was air dried and sieved to 2 mm prior to use. This soil was limed to pH 6.5 based on exchangeable acidic cation content (i.e., H^+^ and Al^3+^) prior to use and was maintained at field capacity. The near-neutral pH was chosen to facilitate the nitrification process. Physicochemical and mineralogical characterization of the soil is discussed in our previous work [13]. Briefly, the soil has a native cation exchange capacity of 7.4 cmol_c_/kg, contains 1.5% organic matter, and has mineralogy dominated by quartz, kaolinite, hydroxyl interlayer vermiculite, gibbsite, hematite, and goethite. Metallic Cu(0) NPs (average particle size: 35 nm, uncoated, % purity: 99.6 ± 0.2, hydrodynamic diameter and zeta potential in 0.01 M NaNO_3_ at pH 6.2: 458 ± 130.3 nm and 13.5 ± 0.7 mV, respectively) were purchased from Nanostructured and Amorphous Materials, Inc. (Houston, TX, USA). For the following experiments, CuNP suspensions were freshly prepared for each use in a 2000 mg/L solution. They were immediately sonified at 25 kHz for 30 s to ensure complete suspension of particles prior to its use. All reagents were prepared with ACS-grade chemicals (Sigma, St. Louis, MO, USA) and Milli-Q distilled, deionized water (18.2 MΩ).

### 2.2. Copper Nanoparticle Dissolution Experiments

Dissolution of CuNPs was conducted under 0.01 M ionic strength (I) using either NaNO_3_ or Na_2_SO_4_. To evaluate the ionic Cu toxicity, one should track the extent of Cu dissolution from the CuNP. The dissolution was assessed in these two electrolytes that were used in this study. It was to investigate the impact of nitrate (i.e., a product of nitrification) during the nitrification experiments. Sulfate was used as the background electrolyte for all analyses to prevent nitrate interference with nitrification analyses. Care was taken to ensure complete suspension of particles in the sample media, including a sonification of a fresh CuNP solution prior to each experiment. Copper NP concentrations ranged from 5 to 1000 mg/L, and solutions were created from stock solutions described above. pH was maintained at 7 ± 0.3, and adjusted with NaOH when necessary. Dissolution experiments were conducted with the initial Cu concentration of 5, 50, and 500 mg/L for 48 h in a batch mode. The dissolution reach pseudo-equilibrium after 48 h. Samples were centrifuged at 28,600× *g* for 29 min to ensure settling of all solid CuNPs before samples were measured for Cu^2+^ concentration using an ion specific electrode (ISE) (Cole-Parmer, Vernon Hills, IL, USA). The detection limit of a cupric ISE was 0.09 mg/L. Ion selective electrodes are known to drift if care is not taken in their storage, maintenance, and calibration. The quality assurance of ISE was performed by minimizing a drift (<3 mM/day). The ISE was calibrated using a series of standards made from a 1000 mg/L standard (EMD Millipore, Burlington, MA, USA) before each measurement. Membranes were polished as needed, and maintained in an appropriate ISE storage solution. Samples were measured for [Cu^2+^] immediately upon collection. Standard curves were limited to two orders of magnitude. The detection limit of the cupric ISE was 0.064 mg/L. A separate set of dissolution experiments were also conducted under the same conditions in the presence of 20 mM 3-Morpholino-2-hydroxypropanesulfonic acid (MOPSO) buffer used for the sorption experiments. Since many organic buffers contain active sites that could function as potential ligands for Cu^2+^, and since dissolution experiments maintained stable pH even without the addition of a buffer, the full suite of dissolution experiments was conducted without MOPSO. Adsorption of ionic Cu on the sidewall of the tubes was negligible (i.e., below the detection limit of Inductively coupled plasma atomic emission spectroscopy (ICP-AES) (SPECTRO Analytical Instruments Inc., Kleve, Germany) after the digestion). The adsorption of CuNPs on the sidewall was included for the final mass balance calculation. The CuNP adsorption on the sidewall of tubes was ≤0.5% of total CuNPs.

### 2.3. Copper Nanoparticle and Cu(II) (aq) Adsorption Experiments

The sorption of whole CuNPs was tested on Toccoa soil in batch mode, using a ratio of 1 g soil to 20 mL solution with a reaction time of 2 days. Since the nitrification experiments were conducted in soil slurry, it was difficult to track the dissolved Cu and CuNPs because of the fast sorption reaction. To assess the adsorption capacity of soils, the batch adsorption experiments were conducted. Copper NPs were added at concentrations ranging from 5 to 1000 mg/L. pH was maintained at 7 (± 0.3 pH units) using 20 mM 3-Morpholino-2-hydroxypropanesulfonic acid (MOPSO) buffer solution, and ionic strength was maintained at 0.01 M using Na_2_SO_4_ salt. All tubes were shaken on an end-over shaker at 16 rpm. After 2d, solutions were centrifuged at 28,600× *g* for 29 min. Stokes’ Law was used to calculate this centrifugation time to ensure that larger soil particles would settle out of solution, but any free CuNPs would remain in solution. Aliquot samples were acidified with HNO_3_ prior to total Cu analysis using ICP-AES. Aliquots without acidification were also tested for potential dissolved Cu^2+^ using an ISE. The difference between total Cu and dissolved Cu^2+^ was used to evaluate the CuNP sorption. Because of the gradual dissolution of metallic CuNP during the nitrification experiments, sorption experiments of Cu^2+^ were also conducted under the same conditions. Cu(II) solutions such as CuSO_4_ were centrifuged at the same rate to maintain consistency and analyzed for [Cu^2+^] and [Cu] total using both ISE and ICP-AES methods, respectively. In the final mass balance calculation, it was assured that there was a negligible contribution from the background Cu in soils. The ICP-AES analysis was performed by the Clemson University Agricultural Service Laboratory. This laboratory follows standard approved analytical methods and procedures, and has comprehensive quality assurance and quality control protocols [20]. A QCS-19 ICP 19 Element Quality Control Standard (High Purity Standards, Inc., Charleston, SC, USA) was used for the quality control. The detection limit of Cu in our sample matrix was 52 µg/L.

### 2.4. Batch Nitrification Kinetic Experiments

The kinetic rate of nitrification was assessed through the oxygenated-shaken slurry method [21]. This slurry method was chosen because it is a well-accepted method in the soil science field and has been already demonstrated in the soil nitrification experiments using the same soil [22]. The maximum nitrification rate, (*V_max_*), is determined over a 24-h period. This value was used as an indicator to estimate the nitrification in these soils.

To maintain bacterial populations, a nutrient solution containing 1 mM NH_4_H_2_PO_4_ and 0.25 mM (NH_4_)_2_SO_4_ was created to ensure that NH_4_^+^ was the limiting nutrient. This solution was maintained at an ionic strength of 0.01 M using Na_2_SO_4_. Each sample was housed in a 125 mL Erlenmeyer flask. To each sample, 50 mL of the solution described above was added to 9 g soil, which had been limed and was maintained at field capacity. Copper NP flasks were dosed with the appropriate amount of freshly-prepared CuNP stock solution to achieve concentrations of 1, 10, or 100 mg/L CuNPs. Ionic Cu flasks were dosed with an appropriate amount of 1000 mg/L CuSO_4_ as Cu^2+^ solution to achieve 1, 10, or 100 mg/L as [Cu^2+^]. Based on preliminary experiments, 100 mg/L Cu^2+^ samples were not feasible as no nitrification was observed. Concentrations of 1 mg/L Cu^2+^ were also avoided as this was not above the background concentration of copper in this soil. Each flask was sealed with vented Parafilm to allow gas exchange, and was placed on an orbital shaker at 180 rpm. This vigorous shaking maintained the oxygenated environment throughout the experiments. 10 mL samples were obtained at 2, 4, 22, and 24 h. At each sampling time, suspensions were centrifuged at 8000× *g* for 8 min, and the supernatant frozen until nitrate analysis was conducted. Each condition was replicated a minimum of eight times to ensure accurate results.

Nitrate was analyzed through a salicylic acid colorimetric technique [23]. Subsamples of 0.80 mL held in 8 mL glass cuvettes were reacted with 0.32 mL of 5% salicylic acid dissolved in sulfuric acid, followed by 7.6 mL of 1.7 M NaOH. After solutions cooled for 30 min, they were measured for absorbance at 420 nm using a spectrophotometer, and concentration was determined using a standard curve. Based on solution volume and soil mass, concentrations were converted to units of mg N/kg soil.

### 2.5. Statistical Analysis of Batch Nitrification Kinetic Experiments

The *V_max_* of each sample was determined from the linear regression analysis of nitrate concentration per kg soil per hour. An overall one-way analysis of variance was conducted to determine whether the variance between groups was greater than the variance within groups. To compare individual conditions, two-tailed *t*-tests were used to determine significant differences unless otherwise mentioned in the text.

### 2.6. X-Ray Absorption near Edge Structure Spectroscopy (XANES) Analysis

To better understand the changes in Cu speciation of Cu(0)NPs in water and soils, XANES analysis was conducted at beamline X11A at National Synchrotron Light Source (NSLS), Upton, NY, USA. The monochromator consisted of two parallel Si (111) crystals with a vertical entrance slit of 0.4 mm and a horizontal entrance slit of 1 cm. Using the CuNP dissolution method, freshly hydrated (~3 h) CuNPs in 0.1 M NaNO_3_ solutions were centrifuged and loaded on a mylar tape. A soil sample was prepared using the batch nitrification method (total Cu as CuNP: 500 mg/kg). After 4 mo under oxic condition, a centrifuged soil sample was loaded on a polycarbonate sample holder covered with a 0.2 mm poly film on the front according to the method described in our previous work (Arai, 2011). The concentration was chosen to meet the detection limit (approximately 500 mg/kg) of XAS measurements at a bending magnet BL X11A at NSLS. The incident X-ray beam was calibrated at 8979 eV, the first inflection point of the first derivative peak of a Cu foil spectrum. The Cu K-edge XANES spectra were collected between 8900 and 9200 eV in fluorescence mode at room temperature using a Ge13 detector. Reference spectra of Cu reference compounds (Cu(I)_2_O, Cu(II)O, and Cu(OH)_2_) (Sigma, St. Louis, MO, USA) and unreacted Cu(0)NP were also collected in transmission mode. All samples were loaded in a N_2_-filled glove bag right before the measurements. Two to three spectra were collected for each soil sample. No beam-induced reduction was observed during the measurements. The XAS data were normalized according to the method described in the previous work [24]. Because of the formation of insoluble Cu compounds, the XANES data were processed using the linear combination of reference compounds to fit the data range of 8920 to 9100 eV. In this analysis, the self-absorption correction function in SIXpack was used and no negative fit [25]. The energy shifts of reference compounds were not allowed during the fit.

## 3. Results and Discussion

### 3.1. Copper Nanoparticle Dissolution Experiments

**Figure 1:**
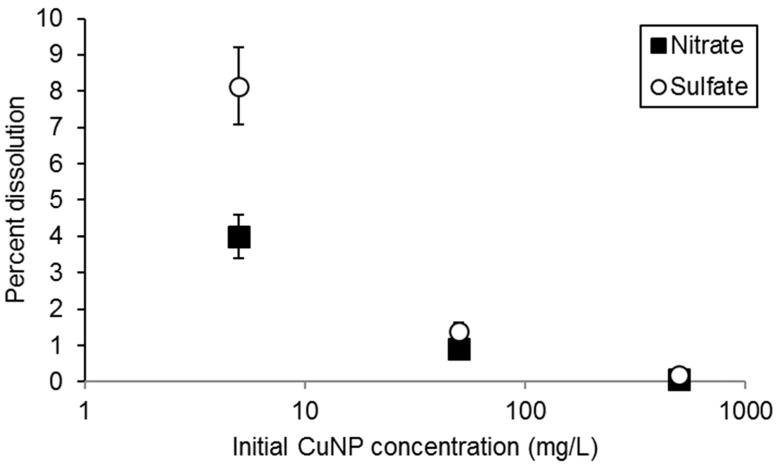
Copper nanoparticle dissolution with sodium nitrate or sodium sulfate background ions to 0.01 M ionic strength. Error bars indicate one standard error above and below the mean. A two-factor analysis of variance indicated significant difference in percent dissolution across both electrolyte (*p* = 0.037) and initial CuNP concentration (*p* < 0.001).

A comparison of dissolution data in two electrolytes is shown in Figure 1. At low concentrations (<10 mg/L), CuNPs displayed maximum dissolution of approximately ~8% (0.4 mg/L) under a sulfate background and approximately 4% (0.2 mg/L) under nitrate background (Figure 1). Under both nitrate and sulfate backgrounds, as the total concentration of CuNPs increases, the percent dissolved Cu decreases predictably. The small difference in dissolution extent between nitrate and sulfate backgrounds only shows a significant difference, as shown by a two-factor analysis of variance, at very high [Cu]_total_ (500 mg/L) concentrations, although it is difficult to see in the % dissolution axis. The sulfate background promoted CuNP dissolution to a greater extent (~0.9 mg/L dissolution) than the nitrate background (~0.35 mg/L dissolution). The extent of CuNP dissolution is important because the release of Cu ions is associated with the production of reactive oxygen species as well as DNA damage in bacteria [26].

It was found that the sulfate ion resulted in slightly heightened Cu^2+^ dissolution from CuNPs at very high [Cu] (Figure 1). A similar trend has been observed by other researchers. In a study of CuO microparticles (<80 μm), 0.5 M H_2_SO_4_ resulted in a higher kinetic rate of dissolution of the particles when compared to the same concentration of HNO_3_, and displayed a lower activation energy for dissolution [27]. With very high [Cu] in the 500 mg/L CuNP experiments, it is likely that these effects are magnified. The effects of sulfate on CuNP dissolution could also be due to the higher affinity of Cu^2+^, a borderline acid on the hard/soft acid/base scale, for sulfur-containing compounds.

Copper and CuO NPs have been reported to have a wide range of dissolution values, indicating that the rate and extent of dissolution is impacted not only by the background ions of the solution, but also by a variety of particle-specific factors such as diameter, production quality, and initial amount of surface-sorbed Cu^2+^ [28]. For this reason, a careful analysis of CuNP sorption is essential in evaluating the toxicity to soil biota. A study by Griffitt et al. [29] found ~25% dissolution of CuNPs (particle size: 80–450 nm, specific surface area: 30.77 m^2^/g) after 48 h at concentrations of 1.25 mg/L [29]. The pattern of diminished Cu^2+^ dissolution with increasing CuNP concentration was noted by Baek and An [30] while studying CuO and other metal oxide NPs, providing evidence of particle-specific factors, rather than simply dissolution, controlling NP toxicity [30]. The relatively low extent of dissolution of these CuNPs, in addition to their toxicity even at low concentrations suggests that the toxicity of these NPs may be due more to whole-particle effects rather than due to the evolution of Cu^2+^ ions from the surfaces of these particles, as also suggested by other researchers studying CuO NPs [30]. This is in contrast to the toxicity pattern displayed by silver NPs under both oxidizing and reducing conditions on the same soils studied here. The AgNPs appear to exhibit toxicity largely based on the amount of Ag^+^ that is dissolved from their surfaces [13,22]. Overall, the CuNPs used in our sorption and nitrification experiments appear to be quite stable, compared to other commercially-available forms.

### 3.2. CuNP and Cu^2+^ (aq) Sorption Experiments

**Figure 2:**
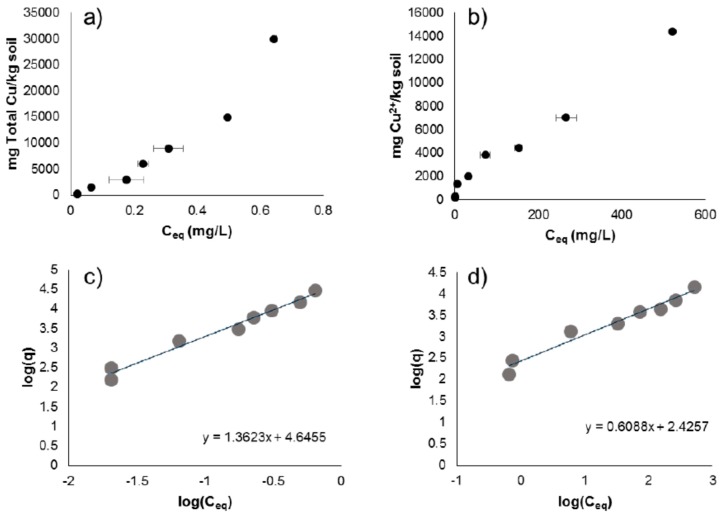
Sorption isotherms of (**a**) CuNPs and (**b**) Cu_2+_ as Cu(II)SO4 onto Toccoa soil. Error bars indicate one standard deviation above and below the Ceq value. Error bars indicating very small standard deviation values are obscured by data markers on some points. Freundlich models for (**c**) CuNPs and (**d**) Cu_2+_ as Cu(II)SO_4_ onto Toccoa soil estimate relative sorptive strength of copper species onto Toccoa soil.

The results of the sorption experiments are shown in Figure 2, which quantifies the amount of Cu sorbed onto soil surfaces compared to the amount of total copper free in solution. All CuNP sorption samples (Figure 2a), when measured for Cu^2+^ on the ISE, were below the detection limit. The instrument was calibrated to measure concentrations as low as which is 0.0006 mg/L. No plateau in CuNP sorption was reached through this experiment. The isotherm displays a nearly linear shape up to C_eq_ = 0.5 mg/L, and the trend became a slightly nonlinear at C_eq_ > 0.5 mg/L.

Ionic Cu^2+^ displayed an S-curve isotherm, as shown in Figure 2b. Again, no plateau in Cu^2+^ sorption was reached through this experiment. Both sorption isotherms were modeled using the pseudo-equilibrium Freundlich model, as shown in Figure 2c,d, and quantified in Table 1. The affinity of CuNP for soil surfaces was extremely large as compared to the affinity of Cu^2+^ for soil surfaces, as indicated by the *K_d_* values for both.

In the presence of soils, the dissolution of CuNPs is different from what is experienced under more controlled aqueous conditions. While Cu^2+^ was measured in all sorption samples using an ISE, the concentration was below detection limit or ISE (detection limit = 0.0006 mg/L Cu^2+^) or no significant concentration was present in soil solution. It is likely that dissolved Cu^2+^ from the CuNP surfaces was adsorbed in soils and or formation of insoluble compounds. In general, metallic NPs exhibit strong sorption onto soil surfaces, or indeed, any solid surface with which they come into contact [13,31]. For this reason, it was not surprising to see the strong sorption of CuNPs onto Toccoa soils (linear range C_eq_ up to 0.5 mg/L in Figure 2). A slightly nonlinear increase at C_eq_ > 0.5 mg/L might indicate the aggregation of CuNPs in soil particles. In our previous AgNPs sorption investigation of the same soils, the high retention capacity of uncoated AgNPs in the same soils was attributed to the sorption of AgNP aggregates in soils [15].

In contrast to the CuNPs, Cu^2+^ displays moderate sorption in the soils (Figure 2 and Table 1). The beginnings of an S-shaped sorption isotherm displayed by Cu^2+^ onto the soil (Figure 2b) is common for Cu^2+^ ion onto mineral soil, as has been noted by other researchers, though it appears that we have not yet reached the sorptive capacity of Cu^2+^ onto this soil. This indicates that while the sorptive capacity of soil organic matter may have been reached, there are additional sites on the mineral components of the soil for Cu^2+^ sorption [32].

### 3.3. XANES Analysis

**Figure 3:**
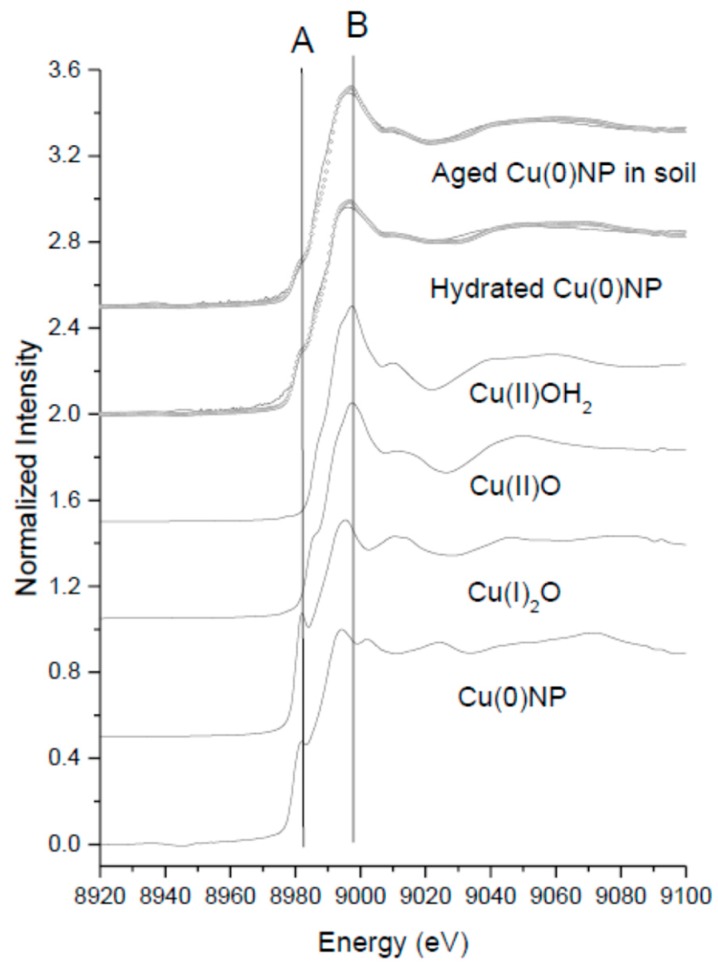
Bulk Cu K-edge NXAFS analysis of aged CuNP reacted soils and Cu reference compounds. Solid lines are normalized raw data and open circles are the fit if LC fit. The results are summarized in Table 2. Vertical lines (A) is aligned at ~8983 eV of the Cu(I) pre-edge peak corresponding to 1s-4p transition. Vertical line (B) is aligned at the absorption peak of Cu(II)O.

In order to better interpret the nitrification potential results below, XANES measurements were conducted to see the potential oxidation of Cu(0) during hydration and the nitrification experiments in soils. It is important to note that XAS spectra of soil without CuNPs were also taken using a GE 13 detector, and the results show negligible edge jump. This suggests that the spectra of CuNP reacted soil represent the Cu speciation of CuNPs in soils. Figure 3 and Table 2 show the results of LC fit XANES analysis of freshly hydrated Cu(0)NPs and aged CuNPs in soils. The results of the LC fit were mainly contributed by CuNPs, Cu(II)O and Cu(OH)_2_, but Cu(I)_2_O. CuSO_4_, CuCl_2_, and Cu(NO_3_)_2_(aq) were also considered in the fit, however, the fit excluded these fractions. It is clear that Cu(0)NPs were contained Cu(I) prior to the hydration (vertical dashed line A in Figure 3). The NPs were further readily oxidized in 0.1M NaNO_3_ to Cu(I) and Cu(II) forming oxide and hydroxide phases. Approximately 51% of the oxidized species were the original CuNPs followed by Cu(OH)_2_, CuO, and Cu_2_O. The partially oxidized CuNPs were further oxidized to Cu(II) in soils. After four months, only 39% of NPs remained, and the rest were Cu(II) species (a vertical line B in Figure 3).

### 3.4. Batch Nitrification Kinetic Experiments

**Figure 4:**
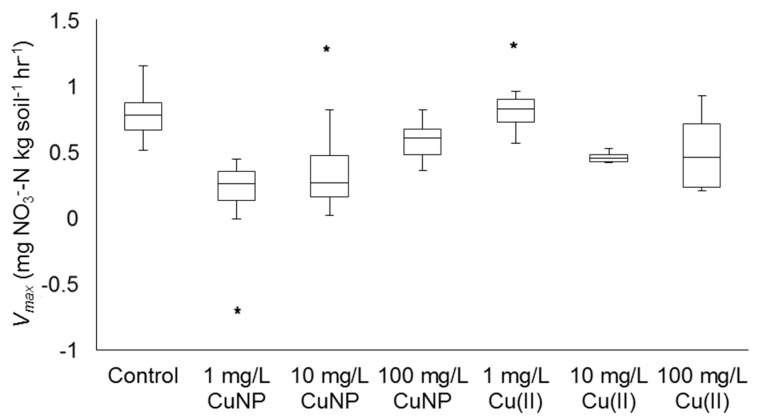
Analysis of variance of nitrification potential (*V_max_*) for each batch nitrification condition. Plots indicate the first and third quartile values at the top and bottom of each box, respectively. Median of each condition is indicated by the line bisecting each box. Error bars indicate the maximum and minimum values within 1.5 times the interquartile range for each condition. Outlier values are indicated by the asterisk (*) symbol. Individual *V_max_* values outside of this range are indicated by stars above or below the box for each condition.

**Figure 5:**
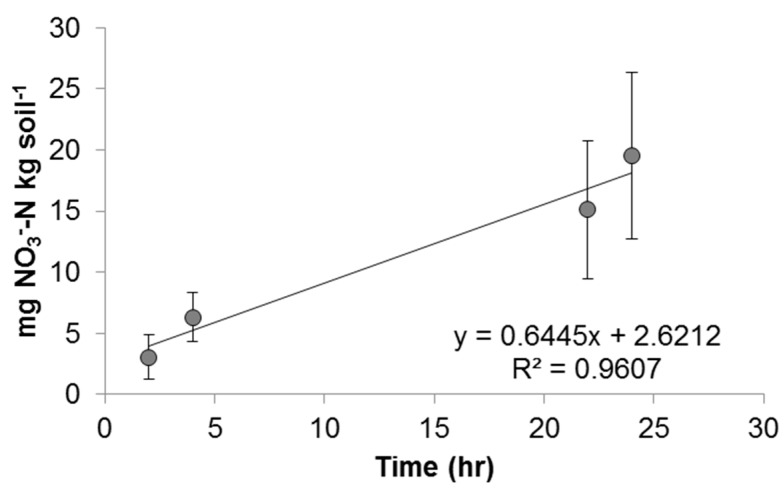
Nitrification kinetics for the control condition. The average *V_max_* is indicated by the slope of the linear regression line, in this case, 0.6445.

**Figure 6:**
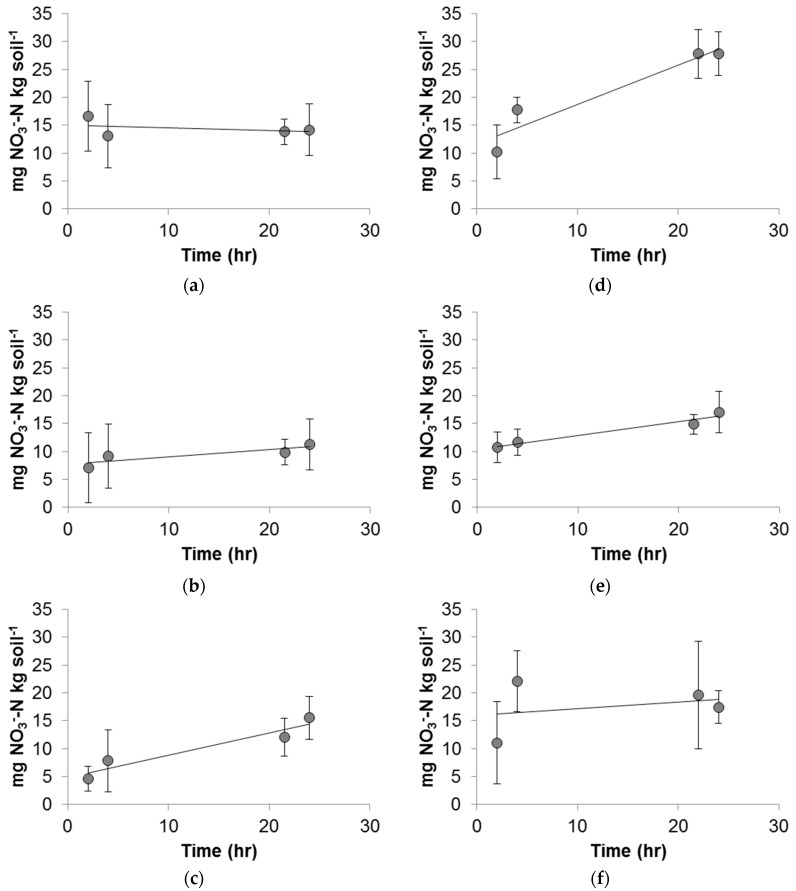
Nitrification kinetics in the presence of various Cu compounds: (**a**) 1 mg/L CuNPs; (**b**) 10 mg/L CuNPs; (**c**) 100 mg/L CuNPs; (**d**) 1 mg/L Cu(II) sulfate as Cu^2+^; (**e**) 10 mg/L Cu(II) sulfate as Cu^2+^; and (**f**) 100 mg/L Cu(II) sulfate as Cu^2+^. Error bars indicate one standard deviation above and below the data point.

The results of a one-way ANOVA showed that the source of variance was largest between conditions, rather than within conditions (*p* < 10^−10^). The variance of each condition is shown in Figure 4. Strong nitrification was observed in the control condition (Figure 5) along with a linear correlation between the concentration of nitrate as N and time, as documented by the high R^2^ value. Experimental conditions ranged from strong, linear nitrification, as in 1 mg/L Cu^2+^ (Figure 6d) to a variable, nonlinear relationship between the concentration of nitrate as N and time, as in 100 mg/L Cu^2+^ (Figure 6f). Full results and *V_max_* values are shown in Figure 6 and Table 3.

Based on the ANOVA results, sources of variance between individual conditions were assessed using two-tailed *t*-tests. All Cu additions, with the exception of 1 mg/L Cu^2+^, negatively impacted the ability of the native soil organisms to complete nitrification in this soil as compared to the control, *p* < 0.05 (Figure 6, Table 4). Due to large variations in results, some conditions were repeated more than the standard eight times (i.e., control and 10 mg/L CuNP). Within the CuNP conditions, the *V_max_* values of 1 and 10 mg/L CuNP conditions were not significantly different from one another, while they were both significantly lower than the control (Table 4). Similarly, within the Cu^2+^ conditions, the *V_max_* values of 10 and 100 mg/L Cu^2+^ did not significantly differ from one another but were both significantly lower than the control (Table 4). The *V_max_* of 100 mg/L CuNP was the highest of all of the CuNP conditions, significantly higher than that of the 1 or 10 mg/L CuNP values. It was significantly lower, however, than the control *V_max_* value. Amongst the CuNP conditions, *V_max_* increased as CuNP concentration increased. Within the Cu^2+^ conditions, *V_max_* decreased as Cu^2+^ concentration increased.

### 3.5. Impacts of Cu^2+^ and CuNPs on Soil Nitrification

To evaluate the effect of CuNPs on the nitrification process, the effects of Cu^2+^ is first discussed because dissolved Cu^2+^ is a dissolution product of CuNP in soils. Because soil experiments generally exhibit some standard deviations in the results, the statistical analysis was carefully conducted using a large group of nitrification experiments. As the XANES analysis indicated, more than 50% of Cu in the NPs is present as Cu(II). At 1 mg/L Cu^2+^, the rate of nitrification kinetics gradually (though not significantly) increased (Figure 5 and Figure 6d, Table 3). This trend supports the importance of Cu as a micronutrient in soils. The nitrifiers were supplied with an essential micronutrient through this addition, instead of a toxicant. Low concentrations of Cu^2+^, under 10 mg/L, have been shown to enhance microbial growth in activated sludge, a community with a strong component of nitrifying bacteria [33]. Copper has also been suggested to provide micronutrient levels of Cu^2+^ to bacteria at low concentrations [34].

However, the toxicity observed from 10 mg/L Cu^2+^ was greater than would be expected from the literature values [35,36]. As shown in Table 3, soils treated with 10 mg/L Cu^2+^ displayed a significantly lower *V_max_* value than the control. At 100 mg/L Cu^2+^, nitrification essentially stopped in some replications and continued at a significantly lower rate in others. This high variability is shown in the varied data shown in Figure 5f and Table 3. The low R^2^ value also indicates high variability and a potentially toxic condition, at least in some replications. This result is to be expected, as high concentrations likely pose toxicity to the vast majority of bacteria in the batch reactor [35,36]. Because of the high concentration, there is no statistical difference in *V_max_* between 10 and 100 mg/L Cu^2+^ (Table 4). To begin to impact the nitrification process, the Cu^2+^ concentration in the soil must be quite large. A 1948 study by Lees documented a 13% inhibition of nitrification when a solution containing 64 mg/L Cu^2+^ was percolated through the soil, also noting that Cu^2+^ showed some toxicity [35]. Similarly, in a more recent study, it was documented that Cu^2+^ required a relatively high concentration (above 250 mg/kg) to cause at least half of the soil bacterial nitrifying community to be affected [36]. In summary, soils treated with ≥10 mg/L Cu^2+^ displayed a significantly lower *V_max_* value than the control.

Through the course of the nitrification experiment, all CuNP conditions exhibited significantly lower *V_max_* values than the control condition (Table 3 and Table 4). The overall negative response of CuNPs is consistent with other observations in the literature (e.g., [34]). In this study, there is no statistical difference in *V_max_* between CuNP 1 and 10 mg/L (Table 4); however, a significant statistical difference was observed between 10 and 100 mg/L systems (Table 4). Concentration independent effects were observed. Shah and Belozerova [5] investigated the impact of CuNPs, among other metallic NPs, on the overall soil microbes in situ, based on the microbial use of various substrates. They found that CuNPs negatively impacted the ability of microorganisms to utilize some commonly available substrates at concentrations of CuNPs as low as 130 mg/kg.

Although we observe the variations in *V_max_* in the CuNP data with respect to the ionic Cu data, the statistical analysis (Table 4) showed that the toxicity of the CuNP is not statistically different from the respective concentration of the ionic Cu^2+^ system. With increasing the concentration of CuNP, what is offsetting the similar toxicological response in the kinetic rate of nitrification, *V_max_*? The concentration of dissolved Cu^2+^ is always greater, though nearly all Cu ions undergo adsorption in soils, in the ionic Cu systems compared to the respective CuNP system. The reasonable explanation is the potential production of reactive oxygen species (ROS) from CuNP. Surface oxidation-enhanced ROS production has been reported by several researchers [26,37]. Although the detection of ROS was difficult in our soil slurry systems because of interference from dissolved organics and other ions in soil filtrates, ROS via the surface oxidation of CuNP cannot be ignored. This could explain the similar suppressed *V_max_* among the same concentration of Cu^2+^ and CuNP.

## 4. Conclusions

This study evaluated the effects of metallic CuNPs as a nanofertilizer on the soil nitrification process. One of the primary drivers for investigating the use of nanoscale Cu is to increase micronutrient delivery and uptake efficacy without causing the negative impact on the soil nutrient cycles. While CuNPs sorb strongly to soils, they showed negative effects on nitrification kinetics between 1 and 100 mg/L. As evident in the XANES analysis, the metallic Cu(0)NP readily oxidized to Cu(II)-oxide and -hydroxides with increasing aging time under oxic condition. The dissolution of Cu^2+^ as well as potential ROS production could explain the suppressed nitrification kinetic rate. The window of [Cu^2+^ (aq)] for beneficial effects as a constituent in soil nitrifier seems very small. The results suggest the delivery of Cu-incorporated nanofertilizer must be carefully evaluated with respect to its trace metal toxicity to microorganisms in various agricultural soils.

## Figures and Tables

**Table 1:** Freundlich equation isotherm parameters for Freundlich models shown in Figure 2c,d. *K_d_* indicated distribution constant for the adsorbent, calculated from the inverse log of the intercept. *n* is the conversion factor, calculated from the inverse of the slope. *p*-values are calculated from the Fisher F statistic at (1, 6) degrees of freedom using the least squares method.

Copper Species	Intercept	*K_d_*	Slope	*n*	R^2^	*p*
CuNP	4.646	44,210	1.362	0.7341	0.9782	<0.001
Cu(II)SO_4_	2.426	266.5	0.6088	1.642	0.9644	<0.001

**Table 2:** The results of linear combination of reference compound fit of Cu K edge X-ray Absorption near Edge Structure Spectroscopy (XANES) spectra shown in Figure 3.

Sample	% Cu(0)NP	% Cu(II)O	% Cu(I)_2_O	% Cu(OH)_2_	Reduced Chi Square
	Bulk XAS Analysis				
Freshly hydrated Cu(0)NPs	51.19 ± 0.5	20.98 ± 0.5	22.4 ± 0.5	6 ± 0.5	0.00101
4mo aged Cu(0)NPs in soil	39.04 ± 0.7	11.03 ± 0.7	-	52.3 ± 0.7	0.00063

**Table 3:** Nitrification kinetics *V_max_* and linear regression values for all conditions. *V_max_* are displayed as ± their standard deviation. *p*-values calculated from the Fisher F statistic at (1, 2) degrees of freedom using the least squares method.

[Cu]_total_ and Cu Species	N	Average *V_max_* (mg NO_3_^−^-N kg Soil^−1^ h^−1^) Value	Average Intercept of Linear Regression	R^2^ of Linear Regression Line	*p*
Control	17	0.645 (±0.190)	2.62	0.961	0.020
1 mg/L Cu^2+^	9	0.703 (±0.236)	11.7	0.908	0.045
10 mg/L Cu^2+^	8	0.247 (±0.0430)	10.4	0.947	0.025
100 mg/L Cu^2+^	8	0.279 (±0.224)	16.0	0.0837	0.683
1 mg/L CuNP	12	−0.0489 (±0.205)	15.1	0.132	0.580
10 mg/L CuNP	20	0.132 (±0.347)	7.68	0.757	0.135
100 mg/L CuNP	13	0.400 (±0.163)	4.90	0.911	0.049

**Table 4:** *p*-values from a *t*-test matrix of average kinetic rate (*V_max_*) for each condition compared to one another. Squares marked “x” indicate a duplicate *t*-test, and are not included.

[Cu]_total_ and Cu Species	Control	1 mg/L CuNP	10 mg/L CuNP	100 mg/L CuNP	1 mg/L Cu^2+^	10 mg/L Cu^2+^
1 mg/L CuNP	0.000285	x	x	x	x	x
10 mg/L CuNP	<0.0001	0.0637	x	x	x	x
100 mg/L CuNP	0.00106	0.00255	0.00588	x	x	x
1 mg/L Cu^2+^	0.534	<0.0001	<0.0001	0.00506	x	x
10 mg/L Cu^2+^	<0.0001	<0.0001	0.565	0.00227	0.00035	x
100 mg/L Cu^2+^	0.0253	0.0424	0.382	0.382	0.0152	0.804

## Abbreviations

CuNP	Copper nanoparticles
ICP-AES	Inductively coupled plasma atomic emission spectroscopy
ISE	Ion selective electrode
MOPSO	3-Morpholino-2-hydroxypropanesulfonic acid
NP	nanoparticles
XAS	X-ray absorption spectroscopy
